# Superoxide dismutase 2 knockdown leads to defects in locomotor activity, sensitivity to paraquat, and increased cuticle pigmentation in *Tribolium castaneum*

**DOI:** 10.1038/srep29583

**Published:** 2016-07-08

**Authors:** Hiroko Tabunoki, Maureen J. Gorman, Neal T. Dittmer, Michael R. Kanost

**Affiliations:** 1 Department of Science of Biological Production, Graduate School of Agriculture, Tokyo University of Agriculture and Technology, 3-5-8 Saiwai-cho, Fuchu, Tokyo 183-8509, Japan; 2 Department of Biochemistry and Molecular Biophysics, Kansas State University, 141 Chalmers Hall, Manhattan, KS 66506-3702, USA

## Abstract

Insects can rapidly adapt to environmental changes through physiological responses. The red flour beetle *Tribolium castaneum* is widely used as a model insect species. However, the stress–response system of this species remains unclear. Superoxide dismutase 2 (SOD2) is a crucial antioxidative enzyme that is found in mitochondria. *T. castaneum* SOD2 (TcSOD2) is composed of 215 amino acids, and has an iron/manganese superoxide dismutase domain. qRT-PCR experiments revealed that TcSOD2 was present through all developmental stages. To evaluate TcSOD2 function in *T. castaneum*, we performed RNAi and also assessed the phenotype and antioxidative tolerance of the knockdown of *TcSOD2* by exposing larvae to paraquat. The administration of paraquat resulted in significantly higher 24-h mortality in *TcSOD2* knockdown larval groups than in the control groups. The *TcSOD2* knockdown adults moved significantly more slowly, had lower ATP content, and exhibited a different body color from the control groups. We found that *TcSOD2* dsRNA treatment in larvae resulted in increased expression of tyrosinase and laccase2 mRNA after 10 days. This is the first report showing that TcSOD2 has an antioxidative function and demonstrates that *T. castaneum* may use an alternative antioxidative system when the SOD2-based system fails.

Insects are exposed to a wide range of environmental stressors, including ultraviolet radiation from sunlight, high or low temperatures, dry conditions/low humidity, lack of food, external hazards, metamorphosis in holometabolous insects, insecticides, and parasitism. These stressors cause the generation of reactive oxygen species (ROS) in the insect’s body^1^. ROS are the intermediates of oxygen reduction processes such as respiration redox reactions, chemical metabolism, and energy production^2^. Under normal conditions, ROS can be useful for the body, as they serve as secondary messengers. However, with excessive exposure to stressors, the production of additional ROS leads to oxidative stress.

Environmental conditions can have physiological effects on insect development, aging, growth, longevity, survival, and reproduction^1^. However, insects can rapidly adapt to environmental changes through physiological responses. For example, the lepidopteran *Bombyx mori* produces colored cocoons that vary from yellow, through pink, golden-yellow, flesh, sasa (yellowish green), and green. The pigments in the yellow, pink, golden-yellow, and flesh cocoons are derived from carotenoids^3^, while those in the sasa and green cocoons are from flavonoids^4^,^5^. These pigments act as antioxidants, protecting the pupae from sunlight^6^. Uric acid (UA), which is the final product of purine metabolism, also plays an important role as a physiological antioxidant^7^. UA accumulates as urate granules in the integument of *B. mori*, causing a whitening of the color, and also accumulates in the wings of *Pieris brassicae*^8^,^9^, protecting the insect’s body against photooxidative stress^10^,^11^. Various types of antioxidant proteins have also been found that regulate the generation of ROS in the body, such as heat shock proteins, superoxide dismutase, DJ-1, and mitogen-activated protein kinase^12^,^13^,^14^,^15^,^16^, all of which are conserved among species. Thus, insects employ the diverse strategies for resistance from environmental stressors. As a result, they are well adapted to environmental change.

The red flour beetle (*Tribolium castaneum*) is a holometabolous insect belonging to the Order Coleoptera. The genome information of this species is well characterized, and systematic RNA interference (RNAi) is available^17^,^18^,^19^,^20^. We were interested in understanding what process *T. castaneum* beetles employ to eliminate ROS from their bodies, as well as the mechanisms by which they adapt to environmental change.

Superoxide dismutase (SOD) is an important antioxidative stress protein that converts the superoxide anion to hydrogen peroxide by acting as a dismutase^21^. Three types of SOD genes have been annotated in the *T. castaneum* genomic database (http://beetlebase.org/): *TcSOD1*, *TcSOD2*, and *TcSOD3*. It has been shown that TcSOD1 responds to some type of stress^22^, and TcSOD3 is also found in *T. castaneum*, although its function remains unclear^23^. It is possible that TcSOD2 might be present in the mitochondrial matrix, as occurs in other species and may respond to oxidative stress^24^. However, the function and characteristics of this protein in *T. castaneum* are currently unclear. The function of SOD2 has been investigated in *Drosophila melanogaster*, where it has been found that flies that are deficient in SOD2 have short life spans, weak tolerance to oxidative stress, and abnormal movements^25^,^26^.

In this study, we investigated the function and characteristics of TcSOD2 using RNAi. We also evaluated the function of TcSOD2 under oxidative stress using paraquat. We found that TcSOD2 has an antioxidative function and that *T. castaneum* may have an alternative antioxidative system that can function when the SOD2-based system fails.

## Results

### Identification and characterization of the TcSOD2 sequence

**Figure 1:**
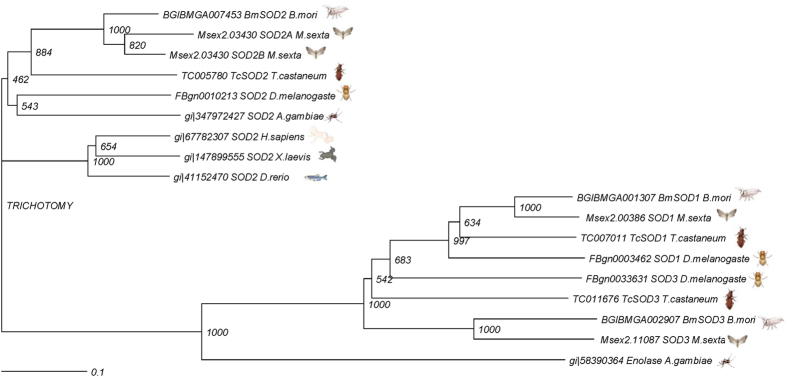
A phylogenetic tree of *T. castaneum* SOD2 and the SOD proteins of other species. The multiple-alignment that was used to construct the phylogenetic tree, and the tree view was then used to display the phylogenetic tree. The insect, animal and baby drawings (http://g86.dbcls.jp/~togoriv/) are licensed under the HYPERLINK http://creativecommons.org/licenses/by/4.0/deed.ja Creative Commons display 4.0 license http://creativecommons.org/licenses/by/4.0/deed.ja.

We obtained the *TcSOD2* sequence from BeetleBase using HMM searches, which yielded one *SOD2* sequence among *T. castaneum* genes (Supplementary Table S1). The sequence was annotated as a manganese superoxide dismutase (MnSOD2) (TC005780) and was localized in ChLG8:9478941…9479642. We confirmed the sequence of TcSOD2 through cDNA cloning, which deduced that the open reading frame was 648 nucleotides long, encoding a protein of 215 amino acids, a molecular weight of 23,642 Da, and a putative isoelectric point of 8.21. TcSOD2 contained an iron/manganese superoxide dismutase alpha-hairpin domain (Sod_Fe_N, pfam; PF00081) at position 18A-99S and an iron/manganese superoxide dismutase C-terminal domain (Sod_Fe_C, pfam; PF02777) at 105P-208R (Supplementary Fig. S1). TcSOD2 showed homology to *B. mori* SOD2 (AB190802, 68%) and *D. melanogaster* SOD2 (FBpp0086226, 62%), *M. sexta* SOD2 (Msex2. 03430-RA, 73%; Msex2. 03430-RB, 70%), and the metal binding amino acid positions were also conserved across these insect species (Supplementary Fig. S1, asterisks). Phylogenetic analysis of TcSOD2, TcSOD1, and TcSOD3 along with the SODs of other insect species placed TcSOD2 in the insect SOD2 cluster (Fig. 1) The nucleotide sequence reported in this paper has been submitted to the GeneBank/DDBJ SAKURA Data bank, Accession No. LC154964.

### TcSOD2 was expressed through all developmental stages

**Figure 2:**
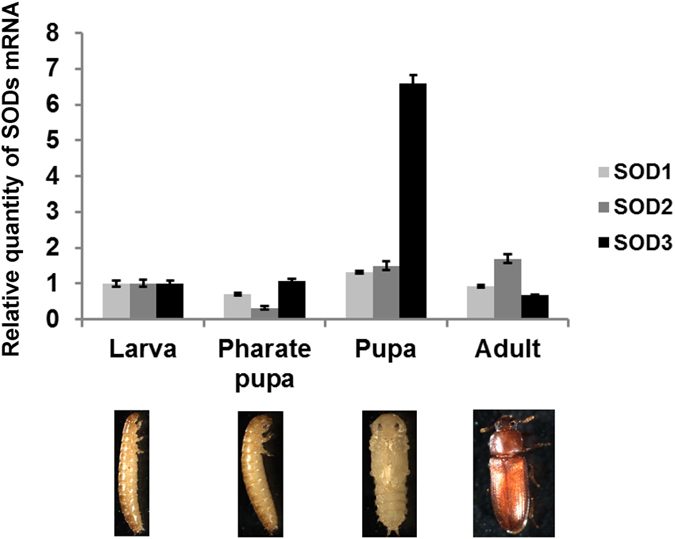
Expression of TcSOD in different developmental stages of *T. castaneum*. Whole bodies were used for qRT-PCR for each developmental stage. Larva, pharate pupa, pupa and adult as Relative Quantification (RQ) values. RQ represents the relative expression level compared to the reference sample. Error bars represent the relative minimum/maximum expression levels about the mean RQ expression level. *TcRpS6* was used as endogenous control.

We examined the mRNA expression of the three types of *TcSOD* genes in four developmental stages using qRT-PCR. All three SODs mRNA were expressed in all of the developmental stages. Expression of TcSOD2 was decreased in pharate pupal developmental stage, while expression of TcSOD3 was increased in pupal developmental stage (Fig. 2).

### Verification of decreased SOD2 mRNA and activity in TcSOD2 knockdown insects

We designed *TcSOD2* dsRNA from cDNA position 1 to 340, and the synthesis of the dsRNA was confirmed by agarose gel electrophoresis. The knockdown effect of this dsRNA was verified by qRT-PCR and measurement of SOD activity. The expression of *TcSOD2* mRNA was decreased in the *TcSOD2* dsRNA-injected groups compared with the *TcVer* dsRNA-injected control groups (Supplementary Fig. S2). In addition, the total SOD activity and SOD2-specific activity (assessed as MnSOD activity) significantly decreased in the *TcSOD2* knockdown group compared with the control group, (Supplementary Fig. S3). Thus, we succeeded to decrease *TcSOD2* mRNA and SOD2 activity. *TcVer* knockdown efficiency was decreased in adult, whereas *TcSOD2* knockdown efficiency was continued in adult (Supplementary Fig. S2).

### Vulnerability of TcSOD knockdown larvae to paraquat-induced oxidative stress

We determined that the LC_50_ of paraquat was lower in *T. castaneum* adults than larvae (adults: 9.86 mM, 95% CI = 7.72–12.43; larvae: 25.20 mM, 95% CI = 17.71–36.71). To assess the oxidative stress resistance ability, we employed paraquat-induced oxidative stress in the *TcSOD2* knockdown larvae and found that the LC_50_ of the *TcVer* knockdown control group was 17.7 mM (95% CI = 9.8–32.2), whereas the LC_50_ of the *TcSOD2* knockdown group was 9.0 mM (95% CI = 3.1–21.7). Thus, the *TcSOD2* knockdown group was tend to be vulnerable to paraquat-induced oxidative stress compared with the control.

### Movement in TcSOD2 knockdown insects

**Figure 3:**
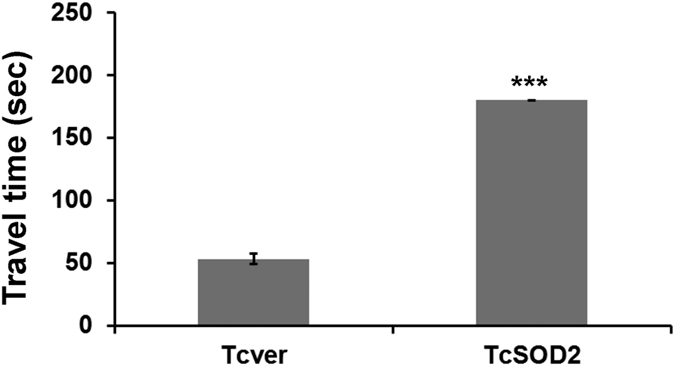
Light-attracted locomotion assay. The *y*-axis shows the travel time (seconds) taken for adults (n = 10 per groups) to move from the bottom of the tube to the cap. The error bars indicate standard deviation (SD). ***P < 0.001 compared with the *TcVer* group (P = 3.26E^−12^).

**Figure 4:**
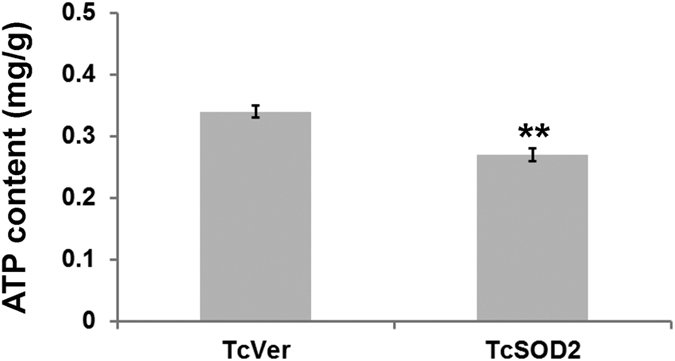
Knockdown of TcSOD2 results in decreased ATP content. dsRNA-treated adult whole bodies of *T. castaneum* were used to measure ATP content (mg/g tissue). The error bars indicate standard deviation (SD). **P < 0.01 compared with the *TcVer* group (P = 0.001).

To evaluate the phenotype of the *TcSOD2* knockdown insects, we observed dsRNA-injected insects. Interestingly, we found that *TcSOD2* knockdown adults showed abnormal movements compared with *TcVer* knockdown adults (Supplementary Videos S1 and S2). These adults not only had a much lower average speed than the *TcVer* knockdown control group (Table 1) but also had a significantly slower running speed, as shown by the light-attracted locomotion assay (Fig. 3). We also found that the ATP content of the *TcSOD2* knockdown insects was significantly lower than the *TcVer* knockdown control group (*P* = 0.001; Fig. 4).

### Other phenotypic changes in TcSOD2 knockdown adults

We observed the survival of *T. castaneum* adults for 60 days and found that the *TcSOD2* knockdown group had significantly lower survival than the *TcVer* control group (Table 2).

**Figure 5:**
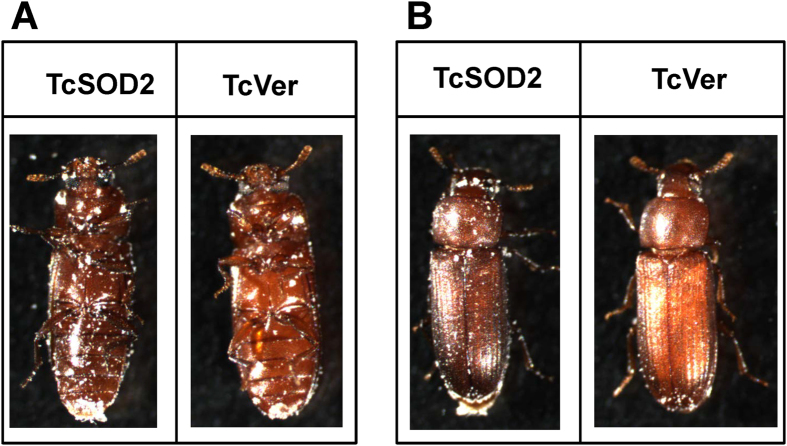
Phenotype of each dsRNA-injected group. (**A**) is ventral view, while (**B**) is dorsal view of each dsRNA-treated adult *T. castaneum*.

**Figure 6:**
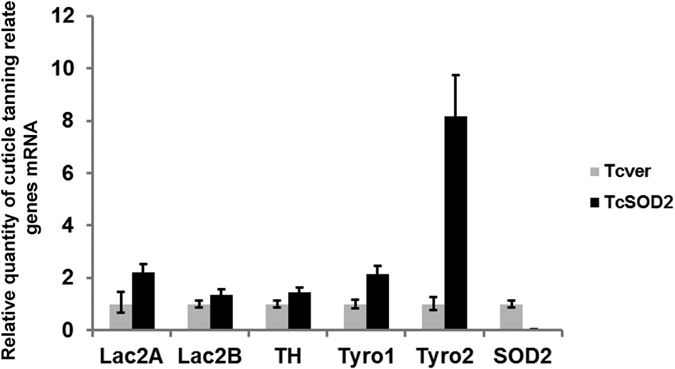
mRNA expression of melanin synthesis-related genes in *TcSOD2* knockdown larvae. The dsRNA-treated whole body was used for qRT-PCR. *TcVer* dsRNA-injected larva and *TcSOD2* dsRNA-injected larva as Relative Quantification (RQ) values. RQ represents the relative expression level compared to the reference sample. Error bars represent the relative minimum/maximum expression levels about the mean RQ expression level. Lac2A, *T. castaneum* laccase 2A; Lac2B, *T. castaneum* laccase 2B; TH, *T. castaneum* tyrosine hydroxylase; Tyro1, *T. castaneum* tyrosinase1; Tyro2, *T. castaneum* tyrosinase2; *TcRpS6* was used as endogenous control.

Unexpectedly, we also found that the body color of *TcSOD2* knockdown adults was dark brown from 1 day after adult ecdysis, in contrast to the reddish brown color of control beetles (Fig. 5). This body color change suggested an increase in melanin in the cuticle. In addition, we observed an increase in expression of tyrosinase 1 and 2, and also in laccase2A, a laccase2 splicing isoform, all potentially involved in oxidation of catechols leading to increased melanin synthesis in the *TcSOD2* knockdown larvae (Fig. 6). Expression of tyrosine hydroxylase, required for production of melanin precursors, was little bit affected by TcSOD2 knockdown.

## Discussion

In this study, we examined the function of TcSOD2 in *T. castaneum.* The metal binding amino acid residues of TcSOD2 were well conserved in the three model insect species investigated, and the phylogenetic tree showed that TcSOD2 is present in the SOD2 cluster, and four insect species SOD2 were closely related.

Paraquat inhibits mitochondrial complex I, leading to generation of ROS^27^,^28^. SOD2 in other species plays a role in removing ROS that have been generated in the mitochondria during ATP production^29^. In this study, the *TcSOD2* knockdown larvae were tend to vulnerable to paraquat-induced oxidative stress, and the *TcSOD2* knockdown adults had a short life span compare to *TcVer* knockdown control group. In addition, we observed that the *TcSOD2* knockdown adults walked and ran much more slowly than the *TcVer* knockdown control group and also had a significantly lower ATP content, consistent with a defect in mitochondrial function.

When we assessed the running speed in the paraquat treatment group with a light-attracted locomotion assay (Supplementary Fig. S4), the running speed of the paraquat treatment group was slower than the control (1% sucrose treatment). Similarly, paraquat treatment of *D. melanogaster* adults resulted in decreased the climbing speed on the vial wall in negative geotaxis assay, because generated ROS caused mitochondrial dysfunction^30^.

Our result showed the *TcSOD2* knockdown beetles were much slower than the Tcver group (Fig. 3). These results are consistent with a hypothesis that *TcSOD2* plays a role in removing ROS that have been generated in the mitochondria and that lack of TcSOD2 results in oxidative stress and impaired mitochondrial function.

A previous study showed that *Sod2* −/− mice (*Mus musculus*) had short life spans (the mean life span was 5.6 days) and experienced cardiomyopathy^31^. Similarly, *SOD2* knockdown *D. melanogaster* have been shown to have short life spans (around 10 days from adult onset), abnormal motion, and a weak tolerance to oxidative stress^32^. While *SOD2* null flies died within 24 hr of adult eclosion and had reduced expression of *SOD1* mRNA^25^. We found that 56.5% of *TcSOD2* knockdown adults died by day 5, but some beetles survived until day 40 (Table 2). Furthermore, the expression of other TcSODs also slightly increased in *TcSOD2* knockdown larvae, and the expression level of TcSOD1 and TcSOD3 was the same in *TcSOD2* knockdown adults as in *TcVer* knockdown adults. Thus, it may be that other SODs compensate for the TcSOD2 function

*SOD2* knockdown *Caenorhabditis elegans* had lower levels of egg production and a weak tolerance to oxidative stress^33^. In *B. mori*, SOD2 may play a role in antioxidative stress tolerance in fifth instar larvae; however, it is not clear whether SOD2 also affects the life span and movement in this species^14^. The function of SOD2 for antioxidative stress tolerance in the mitochondria has apparently been conserved from arthropods to mammals.

Interestingly, we found that *TcSOD2* knockdown adults had a novel phenotype, exhibiting a black-brown color body. Such a phenotypic change has not been found in any previous *SOD2* knockdown study. Sichel *et al*.^34^ previously reported that *Xenopus laevis* livers were stained with melanin, and that SOD1, SOD3, and SOD2 activities were lower in normal animals than in albino mutants. Consequently, they suggested that melanin has a similar role to SODs, because it is able to scavenge superoxide anions, providing the animal with some antioxidative tolerance^34^. Therefore, it is possible that increased melanin synthesis may have been stimulated in the TcSOD knockdown beetles, to serve as an antioxidant by scavenging superoxide anions as an alternative protective response. A black-brown body color in *T. castaneum* might be caused by an increased melanin content^35^,^36^. Since *TcSOD2* knockdown beetles did not exhibit the reduced longevity observed in other *SOD2* knockdown model organisms, this increased melanin content may help to increase their survival time. To further investigate the body color of the *TcSOD2* knockdown adults, we used qRT-PCR to examine the relationship between *TcSOD2* and the following melanin synthesis-related genes: tyrosine hydroxylase, tyrosinase1, tyrosinase2, laccase 2A and laccase 2B. We found that the expression of tyrosine hydroxylase mRNA slightly differ between the *TcSOD2* knockdown group and the *TcVer* knockdown control group. However, the expression of tyrosinase1, tyrosinase2 and laccase 2 A mRNA increased in *TcSOD2* knockdown larvae. Laccase 2A contribute a major cuticle tanning through all developmental stages in *T. castaneum*^36^. Although the role of tyrosinase in cuticle pigmentation is not well established, it is possible that an increased concentration of tyrosinase is responsible for the darker body color of SOD2 knockdown adults.

In conclusion, in this study we examined the function of TcSOD2 and found that it has a conserved antioxidative function across species. We also found that the loss of function of TcSOD2 leads to an alternative, potentially compensating antioxidative system in *T. castaneum*. Therefore, we have elucidated a possible mechanism by which these insects can rapidly adapt to changes in their environmental conditions. In a future study, we will investigate the molecular mechanisms by which TcSOD2 may control melanin synthesis in *T. castaneum*.

## Methods

### Insects

The *T. castaneum* GA-1 strain was used in all experiments. Insects were reared on whole wheat flour containing 5% brewer’s yeast^37^. All insects were kept at 30 °C on a 16-h light/8-h dark cycle.

### Identification of the TcSOD2 sequence by HMM search and Bioinformatics analysis

The hmmsearch program in HMMER (Version 3.1b2)^38^ was used to detect *TcSOD2* candidates. Hidden Markov model (HMM) profiles of the SOD2 N-terminal domain (Sod_Fe_N: PF00081.18) and SOD2 C-terminal domain (Sod_Fe_C: PF02777.14) in the Pfam 28.0 database^39^ were used as queries against protein sequences in the *T. castaneum* Tcas3 Gene Set (ftp://ftp.bioinformatics.ksu.edu/pub/BeetleBase/3.0/) with default parameters.

A search for SOD orthologs in *D. melanogaster* and *B. mori* was conducted using BLAST methods. Global homology searches were conducted using Genetyx ver. 11 (Genetyx Co. Ltd., Tokyo, Japan). Alignment of the deduced TcSOD2 amino acid sequences and SOD homologs from other species and phylogenetic analysis were performed using DDBJ ClustalW 2.0^40^,^41^. The multiple-alignment that was used to construct the phylogenetic tree is presented in Fig. 1. Tree view (http://taxonomy.zoology.gla.ac.uk/rod/treeview.html) was then used to display the phylogenetic tree. Sequences are presented for all SODs obtained from a public database (Supplementary Table S1). A protein motif search was conducted using SMART (http://smart.embl-heidelberg.de/).

### Purification of total RNA and cDNA synthesis from whole-body samples

Whole bodies of larvae, pharate pupae, pupae, and adults (n = 5 per stage) were used for total RNA purification.

These samples were stored at −80 °C until use. These whole bodies were weighed and homogenized with lysis buffer from the PureLink^®^ RNA extraction kit (Thermofisher Scientific Inc., Valencia, CA, USA) and then centrifuged at 13,000 × *g* for 10 min, following which the supernatants were collected and processed for RNA purification according to the manufacturer’s instructions. First, 1 μg of total RNA was treated with DNase I (Invitrogen, Van Allen Way, Carlsbad, CA, USA), and then 500 ng of DNase-treated total RNA was used as template for cDNA synthesis using a PrimeScript™ 1st strand cDNA Synthesis Kit (Takara co. ltd, Tokyo, Japan). Quantitative real time-PCR (qRT-PCR) was performed in 20 μl reaction volumes with 0.125 μl of cDNA template and the specific primers (Supplementary Table 3) with a KAPA SYBR Fast qRT-PCR Kit (Nippon Genetics co, ltd., Tokyo, Japan), in accordance to manufacturer instructions. qRT-PCR was performed on a Step One plus Real-Time PCR System (Applied Biosystems Foster City, CA) following the Delta-Delta Ct method. The *T. castaneum* ribosomal protein S6 gene (*RpS6*, Gene ID 288869507) was utilized as an endogenous reference against which RNA expression levels were standardized, and all data were calibrated against universal reference data. Relative quantification (RQ) values represent the relative expression level against a reference sample. All sample sets were assayed in triplicate as technical replications.

cDNA cloning for TcSOD2 showed in supplementary information section.

### cDNA cloning of TcSOD2 for the synthesis of dsRNA and evaluation of the phenotype

To evaluate the dsRNAs for possible off-target effects, we used the E-RNAi web-service (http://www.dkfz.de/signaling/e-rnai3/). *TcSOD2* dsRNA generated 297 nineteen-nucleotide-long efficient siRNAs that matched *TcSOD2*. However, the E-RNAi identified no off-targets of ds*TcSOD2*. To produce synthesized *TcSOD2* dsRNA, 340 bp of the target site were amplified by PCR using the *T. castaneum* larval cDNA library. Primers are listed in Supplementary Table S4. This fragment was cloned into the PCR vector pMiniT (New England Biolabs, Ipswich, MA, USA) and sequenced.

*T. castaneum* vermillion (*TcVer*, GenBank AY052390) was used as a negative control. *TcVer* dsRNA was synthesized according to the methods of Arakane *et al*.^42^ using the MEGAscript RNAi kit (Ambion), according to the manufacturer’s protocols.

*T. castaneum* larvae (the larva was separated according to size using a No. 25 sieve (Thermo Fisher inc.) were injected with 400–600 ng/200 nl of dsRNAs using a microinjection system (Nanoliter 2000 and Micro4 controller) under a stereomicroscope. We then performed qRT-PCR with the specific primers (Supplementary Table S3 and S5) on the larvae and adults at days 5 and 25 after dsRNA injection, respectively, to assess the knockdown efficiency for the target genes. In addition, we monitored the phenotype of each group over a 2-month period. To evaluate adult survivorship, we counted the number of live insects for 2 months starting from adult day 0 and then calculated the adult survival days.

### Determination of the LC_50_ of paraquat in T. castaneum

To determine the LC_50_ (the concentration at which half of the treated individuals are killed) of paraquat in larval and adult *T. castaneum*, we administered paraquat to larvae (the larva was separated by size with 15 inch serve; n = 10) or adults (n = 20), *TcVer* knock down larvae (n = 5) and *TcSOD2* knock down larvae (n = 5) using the following procedure. These larvae were used 11 days after dsRNA injection. A 1 cm diameter filter paper was placed in a 3.5 cm diameter Falcon culture dish (BD Biosciences, Franklin Lakes, NJ, USA). Each 1 cm filter paper was then saturated with 70 μl of 0, 1.56, 3.125, 6.25, 12.5, 25, 50, or 100 mM paraquat in 1% sucrose solution. The insects were starved overnight before administering paraquat. The number of dead insects after 24 h was counted, and the mortality rate was calculated as % mortality = (*X*/*Y*) × 100, where *X* = the number of dead insects in the group and *Y* = the total number of insects in the group. LC_50_ was calculated by using the Probit Analysis^43^ option in the JMP 10.0 software package (SAS Institute Japan Ltd., Tokyo, Japan).

### Measurement of SOD activity

To assay the SOD activity in insects with a loss of function of *TcSOD2*, an SOD assay kit was used (Dojindo Molecular Technologies, Inc., Rockville, MD, USA). These larvae were used 10 days after dsRNA injection. The whole bodies of larvae (*n* = 3) were homogenized with 200 μl of homogenized buffer (0.25 M sucrose, 10 mM Tris-HCl, pH 7.4, 1 mM ethylenediaminetetraacetic acid (EDTA)) on ice. The homogenate was then centrifuged at 14,000 × *g* for 20 min at 4 °C, following which the supernatant was transferred to a new tube, and total SOD activity was measured. SOD2 activity was measured by adding 100 mM KCN to the homogenate samples (final concentration of KCN = 1 mM).

### Measurement of adenosine triphosphate concentration

Adenosine triphosphate (ATP) was determined using an ATP Bioluminescence Assay Kit CLS II (Roche Applied Science, Mannheim, Germany). The whole bodies of adults that had been injected with each dsRNA after 25 days (*n* = 3) were weighed and then homogenized with 200 μl of boiling 100 mM Tris-HCl and 4 mM EDTA buffer (pH 7.75). The homogenate was incubated for 2 min at 100 °C and then centrifuged at 14,000 × *g* for 10 min at room temperature, following which the supernatant was transferred to a new tube, and ATP was measured according to the manufacturer’s protocols. The emission of luciferase was detected by a multiple wavelength plate reader (Molecular Devices Japan Inc., Tokyo, Japan). The ATP concentration was then determined using standard curve data.

### Protein assay

The total protein concentration was determined using a Micro BCA Protein Assay Kit (Thermo Scientific, Rockford, IL, USA).

### Light-attracted locomotion assay and evaluation of speed

*T. castaneum* adults that had been injected with each dsRNA after 25 days (*n* = 10) were placed on the bottom of a 14-ml Falcon culture polystyrene tube (BD Biosciences) in a dark room, following which the tube was immediately laid down and fixed to the surface using double-sided tape. These adults were attracted to a light that was shone on the side of the cap of the tube (Supplementary Video S3). We measured the time that *T. castaneum* adults took to travel from the bottom of the tube to the cap and repeated this five times per group. Any individuals that showed aberrant behaviors (mating or did not move) were omitted from the dataset. The movement of the adults were also recorded by video, following which their walking speed was analyzed using Image J with wrMTrck plugin^44^.

## Additional Information

**How to cite this article**: Tabunoki, H. *et al*. Superoxide dismutase 2 knockdown leads to defects in locomotor activity, sensitivity to paraquat, and increased cuticle pigmentation in *Tribolium castaneum*. *Sci. Rep.* **6**, 29583; doi: 10.1038/srep29583 (2016).

## Supplementary Material

Supplementary Information

Supplementary video S1

Supplementary video S2

Supplementary video S3

## Figures and Tables

**Table 1:** Walking speed of dsRNA-injected adult *T. castaneum.*

Target for dsRNA	Walking speed (pixels per second)
*TcVer*	52.3 ± 20.3
*TcSOD2*	12.0 ± 6.7***

Mean ± SD walking speed.

***P < 0.001 compared with the *TcVer* knockdown control group (P = 0.00095).

TcSOD, *T. castaneum* superoxide dismutase.

**Table 2:** Effect of TcSOD2 knockdown on the adult life span of *T. castaneum*.

Survival beetle (%)					
Group	~5 days	~10 days	~20 days	~40 days	60 days
TcVer	100	100	100	100	100
TcSOD2	56.5	39.1	34.8	4.3	0

Larvae were treated with TcSOD2 or TcVer dsRNA. The number of live insects was counted from adult day 0 for a 60 days period. Each value shows the percent (%) of survival days for each group.
