# Supplementary material for: Superoxide dismutase 2 knockdown leads to defects in locomotor activity, sensitivity to paraquat, and increased cuticle pigmentation in Tribolium castaneum
Source: Sci Rep. 2016 Jul 8;6:29583. doi: 10.1038/srep29583 (PMC4937408; doi:10.1038/srep29583)

Superoxide dismutase 2 knockdown leads to defects in locomotor activity, sensitivity to paraquat, and increased cuticle pigmentation in *Tribolium castaneum*

Hiroko Tabunoki[1,2,*], Maureen J. Gorman[2], Neal T. Dittmer[2] and Michael R. Kanost[2]

[1] Department of Science of Biological Production, Graduate School of Agriculture, Tokyo University of Agriculture and Technology, 3-5-8 Saiwai-cho, Fuchu, Tokyo 183-8509, Japan.

[2]Department of Biochemistry and Molecular Biophysics, Kansas State University, 141 Chalmers Hall, Manhattan, KS 66506-3702, USA

*To whom correspondence should be addressed. E-mail:h_tabuno@cc.tuat.ac.jp

Availability of supporting data

Supplementary Video S1. *TcSOD2* knockdown movement.

Supplementary Video S2. *TcVer* knockdown movement.

Supplementary Video S3. Light-attracted locomotion assay.

Supplementary Figure S1. Alignment of *T. castaneum* SOD2 with other SOD proteins. The alignment of insect SOD2 amino acid sequences. The conserved metal binding-related amino acids H (His), E (Glu), and D (Asp) are marked by red asterisks (*), while the conserved amino acid residues among species are indicated by an asterisk or colon below the sequences.

Supplementary Figure S2. Verification of the mRNA expression of *TcSOD* by qRT-PCR in *TcSOD2* knockdown *T. castaneum*. The dsRNA-treated whole body was used for qRT-PCR. (A) *TcVer* dsRNA-injected larva and *TcSOD2* dsRNA-injected larva (B) *TcVer* dsRNA-injected adult and *TcSOD2* dsRNA-injected adult as Relative Quantification (RQ) values. RQ represents the relative expression level compared to the reference sample. Error bars represent the relative minimum/maximum expression levels about the mean RQ expression level.

Supplementary Figure S3. Measurement of SOD activity in *TcSOD2* knockdown *T. castaneum*. Extracts of larvae were used to measure SOD activity. (**A**) Total SOD activity (P = 0.006) and (**B**) MnSOD (SOD2) activity (P = 0.0005). The error bars indicate standard deviation (SD). **P < 0.01 and ***P < 0.001 compared with the *TcVer* group.

Supplementary Figure S4. Light-attracted locomotion assay. Ten *Tribolium castaneum* adults were used in this assay, which were treated with a 1% sucrose (control) or an 4.65mM (LC_{25} dose) of paraquat dissolved in 1% sucrose. The y-axis shows the travel time (seconds) that adult *T. castaneum* took to move from the bottom of the tube to the cap. The error bars indicate standard deviation (SD). *P < 0.05 compared with the control group (CNT), P=0.025.

Supplementary Table S1. SOD sequence ID obtained from BeetleBase

SOD name	Position	BeetleBase ID
TcSOD1	ChLG4:13586289…13586750	TC007011
TcSOD2	ChLG8:9478941…9479642	TC005780
TcSOD3	ChLG9:20121715…20122308	TC011676

TcSOD, *T. castaneum* superoxide dismutase.

Supplementary Table S2. Gene ID were used the phylogenic tree in this study.

Species	Product	Gene ID
D.melanogaster	SOD1	FBgn0003462
D.melanogaster	SOD2	FBgn0010213
D.melanogaster	SOD3	FBgn0033631
T.castaneum	SOD1	TC007011
T.castaneum	SOD2	TC005780
T.castaneum	SOD3	TC011676
B.mori	SOD1	BGIBMGA001307
B.mori	SOD2	BGIBMGA007453
B.mori	SOD3	BGIBMGA002907
M.sexta	SOD1	Msex2.00386-RA

M.sexta	SOD2	Msex2.03430-RA
M.sexta	SOD2	Msex2.03430-RB
M.sexta	SOD3	Msex2.11087-RA
H.sapiens	SOD2	67782305
H.sapiens	SOD2	67782307
D.rerio	SOD2	41152470
A.gambiae	SOD2	347972427
A.gambiae	Enolase	58390364
X.laevis	SOD2	147899555

Supplementary Table S3. SOD primers used in reverse transcriptase-PCR

Gene name	Forward	Reverse
TcRpS6	5′-GGACCCAAAAGAGCATCAAA-3′	5′-CCTCAAGCAACGCTTCTTCT-3′
TC007011_SOD1	5′-GGTGATGGAGTTGCCAAAGT-3′	5′-GATTCCAACAACACCACACG-3′
TC005780_SOD2	5′-CAAGTCCGGGAAATTGAAAA-3′	5′-CTCGAAAATGGCCTTGACAT-3′
TC011676_SOD3	5′-CTTCCACATCCACGAGAAGG-3′	5′-CACCACAGCCCTCCCTATAA-3′
TcVer	5′-CGTAATGAGTTGCCCACTGAG	5′-CGACTCTTCTAAAACGTCGCT

	AC-3′	GA-3′

SOD, superoxide dismutase.

Supplementary Table S4. dsRNA synthesis primers used in this study

Target gene	Forward	Reverse
*TcSOD2_dsR*NA_target site	5′-TGTCAAACGTCAAGGTT-3′	5′-GAGCGCCGGTTCGAGGG-3′
*TcSOD2_dsR*NA_target site With T7	5′-TAATACGACTCACTATAGGGTGTCAAACGTCAAGGTTA-3′	5′-TAATACGACTCACTATAGGGGAGCGCCGGTTCGAGGGA-3′
TcVer with T7	5′-TAATACGACTCACTATAGGGCCGCCATTTC-3′	5′-TAATACGACTCACTATAGGGGTCTTGGTGGA-3′
T7	5′-TAATACGACTCACTATAGG-3′	5′-TAATACGACTCACTATAGG-3′

TcSOD, *T. castaneum* superoxide dismutase.

Supplementary Table S5. Melanin synthesis-related gene primers used in reverse transcriptase-PCR

Gene name	Forward	Reverse
TcTH	5’- AGACCCCATTCCCTACATCC-3’	5’- GGTGTGGCGTTTCAAAAAGT-3’
TcLac2A	5’- TATCTACACGCCTGAGGATC-3’	5’- TCGAGTTAATTTCTGGCACG-3’
TcLac2B	5’-AAACCCGAAGACATTTTCCG-3’	5’- GATTGTCCGCTCGAAATCTG-3’
TcTyro_1	5’-AGATTCGCCTGAGTCTGGAC-3’	5’-GCTTGCACCTCGATGTTGGT-3’
TcTyro_2	5’-GACGTGGAAGACTTGAAGAGG-3’	5’-CACGTGCCTTTCCTATTTCCG-3’

Supplementary Material and Method

cDNA cloning for TcSOD2- The ORFs of TcSOD2 was amplified by PCR using KOD DNA polymerase (TOYOBO, Tokyo, Japan) with the following primers: 5’-TGTCAAACGTCAAGGTTAGA-3 and 5’-CGCAAATGAATTACAGTACG-3. Product was reacted with 10x Attachment mix (TOYOBO) and cloned into pCR^{TM}II-TOPO vector using a TOPO TA cloning kit (Invitrogen). The nucleotide sequence was confirmed by DNA sequencing.

Supplementary Figure S1

```
                                        *
Msex2.03430_SOD2A_M.sexta           -MFNLN--LFAIHSMYIYRIS----------------------------- 18
Msex2.03430_SOD2B_M.sexta           MLINNY--FVALPWMFSWSLSRTVGASRQKHTLPELPYEYSALEPVINRE 48
BGIBMGA007453_BmSOD2_B.mori         MLMSQR--IGSLIRVAGASRQ--------KHTLPELPYEYNALEPVISRE 40
TC005780_TcSOD2_T.castaneum         -MFALR--RLATAPLAGSRAA---------HSLPELPYAYEALEPVISRD 38
FBgn0010213_SOD2_D.melanogaster     -MFVAR--KISQTASLAVRGK---------HTLPKLPYDYAALEPIICRE 38
gi|347972427_SOD2_A.gambiae         -MLAVRGALFSTAKNCSAVLG-----CRSKHTLPDLPYDFGALEPVICRE 44
                                     ::       :

                                        *
Msex2.03430_SOD2A_M.sexta           LICIFY---------------------VKGDIDTIINLAPALKFNGGGH 46
Msex2.03430_SOD2B_M.sexta           IMSLHHSKHHATYVNNLNAAEEKLAQAQSKGDIDTIINLAPALKFNGGGH 98
BGIBMGA007453_BmSOD2_B.mori         IMSLHHSKHHATYINNLNVAEEKLAQAQAKGDIDTIINLAPALKFNGGGH 90
TC005780_TcSOD2_T.castaneum         IMCLHHSKHHQTYVTNLNAAEEKLKAALSKGDISTAISLEPALRFNGGGH 88
FBgn0010213_SOD2_D.melanogaster     IMELHHQKHHQTYVNNLNAAEEQLEEAKSKSDTTKLIQLAPALRFNGGGH 88
gi|347972427_SOD2_A.gambiae         IMELHHQKHHNAYVTNLNAAEEQLQDAVAKQDVSKIIQLGNAIKFNGGGH 94
                                    :: :.:                       * *  .  *.*  *::******

                                      *
Msex2.03430_SOD2A_M.sexta           INHTIFWQNLSPNGGKPSDALTKAVEKDFGSWENMKNQLANSSVAVQGSG 96
Msex2.03430_SOD2B_M.sexta           INHTIFWQNLSPNGGKPSDALTKAVEKDFGSWENMKNQLANSSVAVQGSG 148
BGIBMGA007453_BmSOD2_B.mori         INHSIFWHNLSPNGGKPSDVLTKAVEKDFGSWDNIKNQLSTASVAVQGSG 140
TC005780_TcSOD2_T.castaneum         LNHSIFWQNLSPESTQPSDALKKAIEDSFGGVQQLKDQLSASSIGVQGSG 138
FBgn0010213_SOD2_D.melanogaster     INHTIFWQNLSPNKTQPSDDLKKAIESQWKSLEEFKKELTTLTVAVQGSG 138
gi|347972427_SOD2_A.gambiae         INHSIFWKNLSPDRSDPSAELQKALNRDFQNMENFKKEMKAAAVAVQGSG 144
                                    :**:***:****:  .**  * **:: .: . :::*.::   ::.*****

                                                                        *  *
Msex2.03430_SOD2A_M.sexta           WGWLGYNKQMKKLQIATCQNQDPLQATTGLVPLFGIDVWEHAYYLQYKNV 146
Msex2.03430_SOD2B_M.sexta           WGWLGYNKQMKKLQIATCQNQDPLQATTGLVPLFGIDVWEHAYYLQYKNV 198
BGIBMGA007453_BmSOD2_B.mori         WGWLGYNKQMKKLQIATCQNQDPLQATTGLVPLFGIDVWEHAYYLQYKNV 190
TC005780_TcSOD2_T.castaneum         WGWLGYCSKSGKLKIATCANQDPLQATTGLVPLLGIDVWEHAYYLQYKNV 188
FBgn0010213_SOD2_D.melanogaster     WGWLGFNKKSGKLQLAALPNQDPLEASTGLIPLFGIDVWEHAYYLQYKNV 188
gi|347972427_SOD2_A.gambiae         WAWLGYNKKTKLLQIAACPNQDPLEATTGLVPLLGIDVWEHAYYLQYKNL 194
                                    *.***: .:   *::*:  *****:*:***:**:***************:

Msex2.03430_SOD2A_M.sexta           RADYVKAIFDVANWNDVSARFEKALK--- 172
Msex2.03430_SOD2B_M.sexta           RADYVKAIFDVANWNDVSARFEKALK--- 224
BGIBMGA007453_BmSOD2_B.mori         RADYVKAIFDVANWNDISQRYEKALK--- 216
TC005780_TcSOD2_T.castaneum         RADYVKAIFEIVNWKDVSQRFEKAKSC-- 215
FBgn0010213_SOD2_D.melanogaster     RPSYVEAIWDIANWDDISCRFQEAKKLGC 217
gi|347972427_SOD2_A.gambiae         RPNYVDAIFDVVNWKDVSERLAKAH---- 219
                                    *..**.**:::.**.*:* *  :*
```

Supplementary Figure S2

A

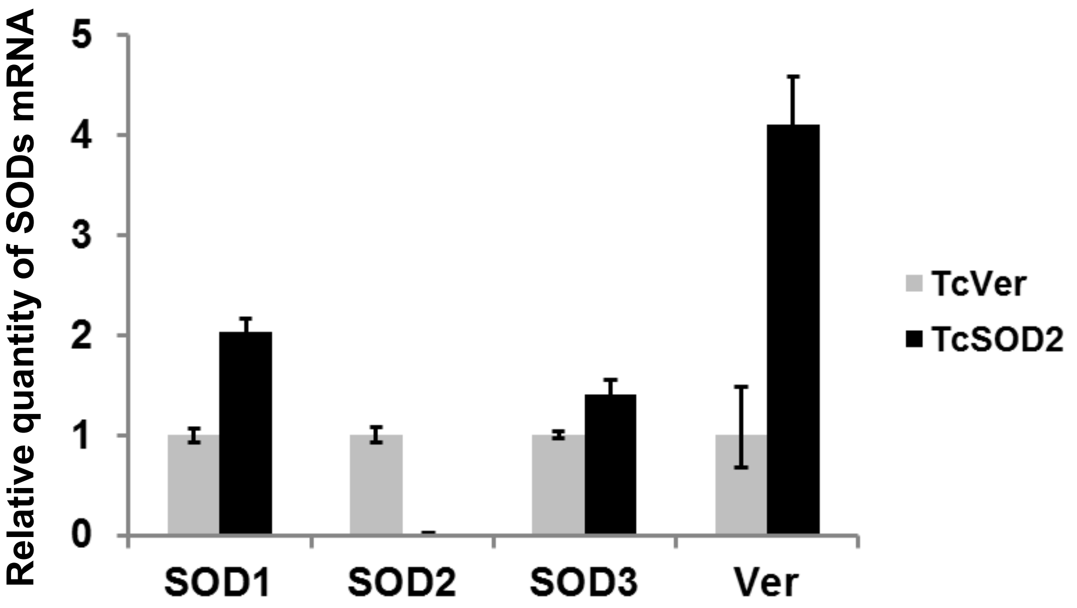

B

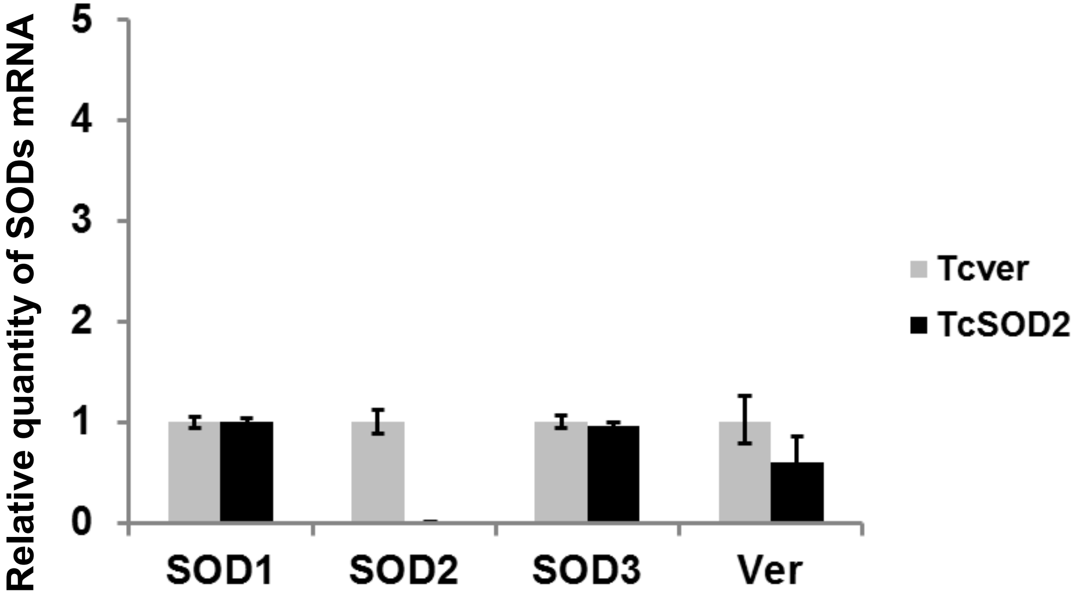

Supplementary Figure S3

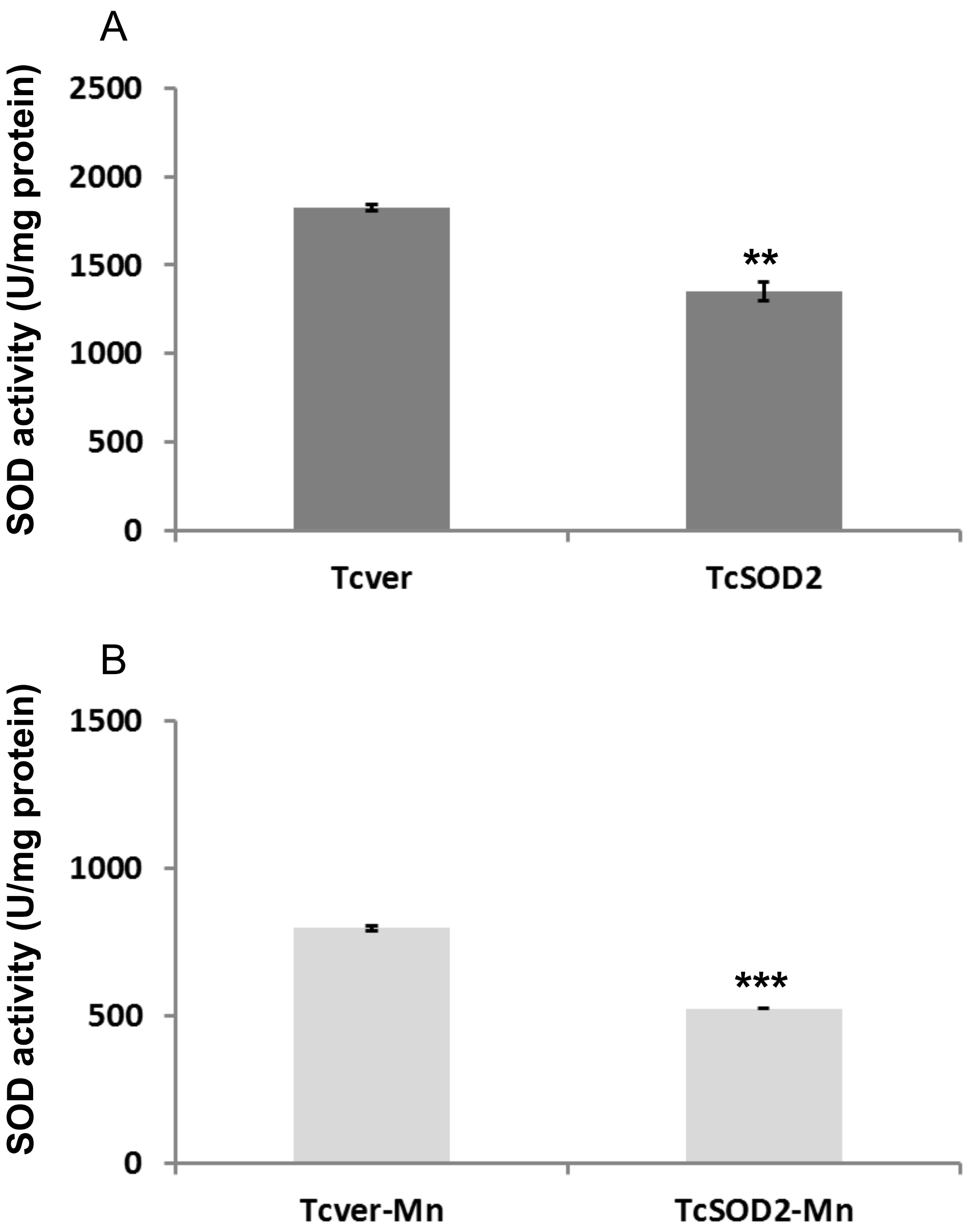

Supplementary Figure S4

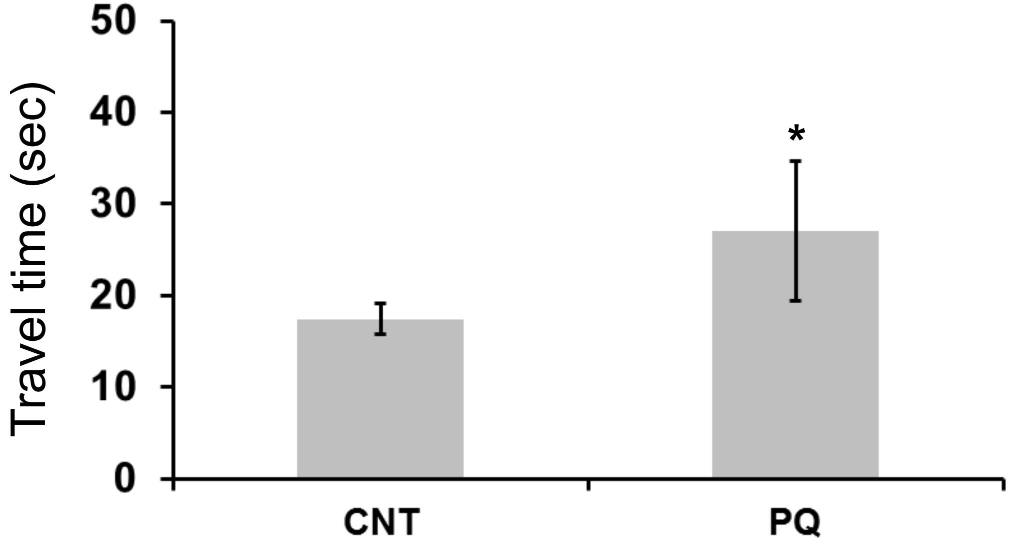

Supplement: Supplementary Information [file srep29583-s1.pdf]
